# Design and Development of Natural-Product-Derived Nanoassemblies and Their Interactions with Alpha Synuclein

**DOI:** 10.3390/biomimetics10020082

**Published:** 2025-01-28

**Authors:** Ipsita A. Banerjee, Amrita Das, Mary A. Biggs, Chau Anh N. Phan, Liana R. Cutter, Alexandra R. Ren

**Affiliations:** Department of Chemistry and Biochemistry, Fordham University, 441 East Fordham Road, Bronx, NY 10458, USAmbiggs2@fordham.edu (M.A.B.); cphan8@fordham.edu (C.A.N.P.); lcutter@fordham.edu (L.R.C.); aren6@fordham.edu (A.R.R.)

**Keywords:** alpha synuclein, terpenes, self-assembly, cellular uptake, nanoassemblies

## Abstract

Biomimetic nanoassemblies derived from natural products are considered promising nanomaterials due to their self-assembling ability and their favorable interactions with biological molecules leading to their numerous applications as therapeutic agents or as molecular probes. In this work, we have created peptide nanoconjugates of two natural products, β-Boswellic acid (BA) and β-glycyrrhetinic acid (GH). Both BA and GH are known for their medicinal value, including their role as strong antioxidants, anti-inflammatory, neuroprotective and as anti-tumor agents. To enhance the bioavailability of these molecules, they were functionalized with three short peptides (YYIVS, MPDAHL and GSGGL) to create six conjugates with amphiphilic structures capable of facile self-assembly. The peptides were also derived from natural sources and have been known to display antioxidant activity. Depending upon the conjugate, nanofibers, nanovesicles or a mixture of both were formed upon self-assembly. The binding interactions of the nanoconjugates with α-Synuclein, a protein implicated in Parkinson’s disease (PD) was examined through in silico studies and FTIR, circular dichroism and imaging studies. Our results indicated that the nanoassemblies interacted with alpha-synuclein fibrils efficaciously. Furthermore, the nanoassemblies were found to demonstrate high viability in the presence of microglial cells, and were found to enhance the uptake and interactions of α-Synuclein with microglial cells. The nanoconjugates designed in this work may be potentially utilized as vectors for peptide-based drug delivery or for other therapeutic applications.

## 1. Introduction

Over the years, natural product-based biomimetic nanoscale assemblies are gaining importance due to their plethora of biological and biomedical applications [1,2]. For example, the plant-derived flavonoid dihydromyricetin has been utilized to prepare nanoassemblies that showed strong antioxidant activity and distribution within the GI tract and were utilized for treatment of Inflammatory Bowel Disease (IBD) [3]. In another study, self-assembled curcumin-erlotinib conjugated nanoparticles were found to exhibit high potency in targeting pancreatic tumor cells and mitigated cell invasion in mice xenograft models [4]. Amongst phytochemicals, in addition to alkaloids, flavonoids and non-flavonoid polyphenols, terpenoids are another class of compounds that have garnered importance due to their anti-inflammatory, antimicrobial, antioxidant or anti-tumor activity [5,6]. For instance, self-assembled nanoparticles of berberine and methoxycinnamic acid have shown potent activity as anti-microbials [7]. The pentacyclic triterpenoid glycyrrhetinic acid (GH) derived from licorice has been found to inhibit liver fibrosis, and demonstrated antioxidant effects in mice [8]. Furthermore, nanoliposomes of GH encapsulated with branched-chain amino acids such as isoleucine and valine as well as with glutamine and arginine were found to reduce cisplatin-induced liver toxicity [9]. In addition, nanovesicles of terpenoids such as D-Limonene and 1,8-cineole have been utilized for dermal drug delivery [10].

Specifically, terpenoids contain varying isoprene units that are comprised of a lipophilic backbone as well as polar functional groups that promote self-assembly due to intricate intra and intermolecular interactions [11]. For example, the dihydroxy triterpenoid betulin derived from the bark of white Birch trees and α-onocerin isolated from *Lycopodium clavatum* can spontaneously self-assemble into nanoflower-like morphologies [12,13]. To further enhance the efficacy of terpenoids, they have been conjugated to fatty acids, fluorescent dyes or peptides. For example, the sesquiterpene Thapsigargin, known for its ability to inhibit sarcoplasmic/endoplasmic reticulum (ER) calcium ATPase (SERCA), has been conjugated with the fluorescent probe BODIPY. The conjugate was found to be localized in SERCA-like calcium pump in the protozoan Trypanosoma evansi [14,15] and derivatives of Thapsigargin have since been considered for drug development. In addition, naturally occurring terpenes conjugated to glycosidic moieties have been found to be applicable in targeting neurodegenerative diseases. For instance, the ginseng extract ginsenoside Rg3, a triterpenoid, has been shown to reduce the formation of beta-amyloid fibrils in CHO2B7 cells and in transgenic mice, and therefore may have implications in possibly targeting Alzheimer’s disease [16,17]. Furthermore, tri-terpenoids including tenuifolin and oleanolic acid have been found to show neuroprotective effects and are beneficial for potentially mitigating symptoms of Alzheimer’s disease [18,19]. In a recent study, the pentacyclic triterpenoid Boswellic acid and other extracts of Boswellia were found to reduce the loss of dopaminergic neurons and mitigated the accumulation of α-Synuclein caused by rotenone [20]. Furthermore, several studies in animal models have demonstrated the anti-inflammatory and antioxidant activity of Boswellic acid (BA) [21,22]. Studies have also shown that beta-Boswellic acid can synergistically work with poly ε-caprolactone (PCL)–gelatin scaffolds and induce dopaminergic differentiation of CGR8 embryonic stem cells, and may be utilized in neural tissue engineering applications [23]. In a recent study, gold nanoparticles were conjugated with β-Boswellic acid and those hybrid nanoparticles were found to inhibit tau protein aggregation and prevented the formation of tau fibrils, and therefore may be applicable in the potential treatment of Alzheimer’s disease [24]. In another study, drug-loaded nanoparticles of PLGA (poly lactic-co-glycolic acid) and polyvinyl alcohol (PVA) were prepared by co-precipitation of Boswellic acid with the polymers which demonstrated a reduction in proliferation of HepG-2 cancer cell lines [25]. Additionally, BA-coated zinc nanoparticles have been prepared which were found to specifically reduce NF-κB-mediated inflammation caused by dextran sodium sulfate (DSS)-induced ulcerative colitis in rats [26]. In addition, derivatives of glycyrrhetinic acid (GH) containing 1,3 diaminopropyl or 1H-benzotriazolyl moieties have been developed to selectively inhibit the activity of butyrylcholinestarase (BChE) enzyme as a strategy toward targeting Alzheimer’s disease. Furthermore, those derivatives showed strong binding interactions with both acetylcholinesterase (AChE) and BChE enzymes through in silico molecular docking studies [27]. Sohn and co-workers demonstrated anti-inflammatory and anti-apoptosis effects of 18-β-GH and indicated their potential applications against brain tissue inflammation [28]. In a separate study, the glycone derivative of GH, glycyrrhizic acid (GZ), has been shown to mitigate amyloidigenesis and fibrillation of proteins such as lysozyme while its diammonium glycyrrhizinate derivative reduced amyloid beta (1–42)-induced inflammation in both in vivo and in vitro studies [29,30]. In their recent pioneering study, Pang and co-workers examined the binding mechanism of GZ with the protein α-Synuclein which was largely attributed to hydrogen bonds with the N-terminal domain and the non-amyloid binding region (NAC) domain of α-Synuclein as well as intermittent binding to the C-terminal domain. All of those were considered as requirements for preventing α-Synuclein aggregation and modulating synucleinopathies [31].

**Figure 1:**
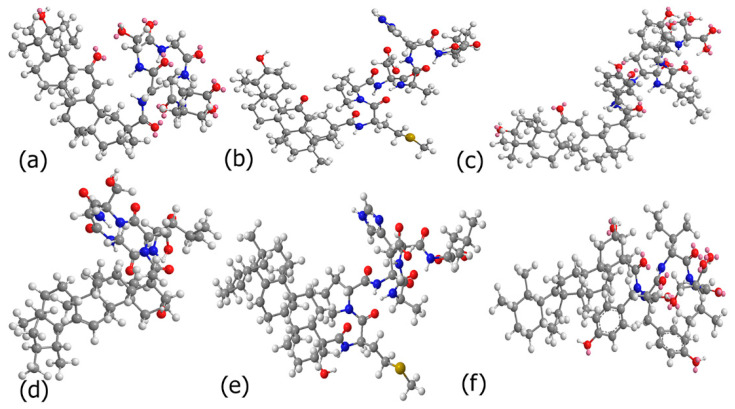
Chemical structures of the designed conjugates. (**a**) Glycyrrhetinate-GSGGL; (**b**) glycyrrhetinate-MPDAHL; (**c**) glycyrrhetinate-YYIVS; (**d**) Boswellate-GSGGL; (**e**) Boswellate-MPDAHL; (**f**) Boswellate-YYIVS. Blue: nitrogen; red: oxygen; grey: carbon; and white: hydrogen.

Given the biologically favorable antioxidant and anti-inflammatory properties of GH and BH, in this study, we created new peptide conjugates of BA and GH and investigated their interactions with the protein α-Synuclein through computational and laboratory studies. The rationale being that peptide conjugates may enhance interactions with α-Synuclein (α-Syn), and thereby aid in enhancing the bioavailability of GH and BA and modulate α-Syn binding. To our knowledge, this is the first time that peptide conjugates of BA and GH have been developed to examine the binding interactions with α-Syn, a protein which has been implicated in Parkinson’s disease (PD) [32]. Furthermore, the peptides utilized for conjugation with BA and GH were short sequences GSGGL, YYIVS and MPDAHL which were previously shown to demonstrate antioxidant activity [33,34]. Thus, combining naturally derived antioxidant terpenes with these antioxidant peptides may also potentially aid in the reduction of reactive oxygen species that cause misfolding of alpha synuclein, leading to mitochondrial dysfunction which is known to exacerbate PD [35]. Additionally, these short peptides contain hydrophilic moieties such as serine or charged residues such as histidine, and aspartic acid. Upon conjugation with the terpenes GH or BA, the conjugates would be rendered amphiphatic lending them to have a higher propensity of self-assembly and formation of nanostructures [36] that may be potentially developed for nanoscale therapeutics. In previous work, we have shown that these peptide sequences in conjunction with the terpene celastrol were found to bind to superoxide dismutase I (SOD I) proteins and modulated their folding process [37]. SOD1 misfolding has been implicated in amyotrophic lateral sclerosis (ALS) disease. Studies have also shown that there may be an association between neurodegenerative diseases such as classical ALS and Parkinson’s disease (PD), and therefore, investigating the effects of these terpene–peptide conjugates of BA and GH may lead to new strategies in the targeting of neurodegenerative diseases [38]. The chemical structures of the conjugates are shown in Figure 1. The binding affinities of the peptides and the conjugates were examined through molecular docking studies. The stability of the complexes was explored by conducting MD simulations. To validate the computational results, the peptide conjugates were synthesized and then self-assembled to form nanoscale assemblies and their interactions with α-Syn were explored. Our results indicated that the nanoassemblies successfully interacted with α-Syn and led to conformation changes. Furthermore, the nanoassemblies were found to be non-cytotoxic and promoted the uptake of α-Syn in microglial cells. Such biomimetic nanoscale assemblies with inherent anti-inflammatory properties may be effective as vehicles for drug delivery for dual targeting or as probes for examining biological interactions or potentially developed for targeted therapeutics through further functionalization.

## 2. Materials and Methods

### 2.1. Computational Methods

#### 2.1.1. Conjugate Design

Each compound (β-Boswellic acid (BA) and 18-β-glycyrrhetic acid (GH)) and their peptide conjugates were designed utilizing Chem Draw (23.1.1). For each peptide conjugate, the N-terminus of the peptide sequence (YYIVS; GSGGL or MPDAHL) was conjugated to the carboxyl group of BA or GH. The three-dimensional structures of each of the peptides and their conjugates were energy minimized using Chem 3D and then saved as PDB files. Those PDB files were then imported into PyMOL (2.5.2).

#### 2.1.2. Binding Pocket Analysis (POCASA)

To determine the surface cavities and the binding regions of alpha-synuclein (α-Syn) fibrils (PDB ID: 6H6B) [39], we downloaded the pdb file from RSCB Protein databank [40]. Any attached ligands and water molecules were removed. The webserver POCASA (POcket-CAvity Search Application) [41], was used to determine surface cavities and clefts within the α-Syn protein. The pdb file was uploaded to the server for analysis. POCASA utilizes an algorithm, “Roll”, to predict the binding regions by detecting pockets and cavities by utilizing a rolling sphere model. Results included output pdb files and analysis parameters including volume depth (VD) and volume of the pockets. We used standard parameters which included a probe radius of two angstroms, an Single Point Flag (SPF) of 16, a Protein-Depth Flag (PDF) value of 18, an unlimited number of cavities and a grid size of 1 A°.

#### 2.1.3. Molecular Docking

Molecular docking studies were conducted using Auto Dock Vina v.1.2.0 [42,43] to determine the optimal binding positions and affinities of the conjugates with the alpha-synuclein fibril structures (PDB ID: 6H6B). The protein was first downloaded from the RCSB Protein Databank in .pdb format and visualized in PyMOL (v.2.5.2) to remove water molecules and ligands, followed by the addition of hydrogen bonds. The modified files were saved as .pdb and imported into AutoDockTools (v.1.5.6), where Kollman charges were added before converting the files to .pdbqt format. The BA–peptide and GH–peptide conjugates, previously designed in ChemDraw 3D, were also opened in AutoDockTools (v.1.5.6) for water molecule removal. A grid was then created to specify the docking region for Auto Dock Vina, with the grid size set at 40 Å × 40 Å × 40 Å for alpha-synuclein fibrils. The grid was centered at x = 116.299 Å; y = 116.273 Å; z = 93.378 Å. Each of the ligand-bound fibril complexes were saved as .pdbqt files and uploaded into Auto Dock Vina v.1.2.0 for docking. The docking poses with the lowest RMSD values were selected as the most optimal structures, and binding affinities were determined in kcal/mol. In addition, to determine if the conjugates had binding specificity toward alpha-synuclein, we also conducted molecular docking studies with four other amyloidigenic proteins, (PDB ID: 7LNA—infectious prion fibrils [44]; PDB ID: 6Y1A—amyloid fibril structure of islet amyloid polypeptide [45]; PDB ID: 8EZE-brain-derived 42-residue amyloid beta fibril type B [46]; and 7UPG—Tau paired helical filament from Alzheimer’s disease [47] using the same methodology as described for the alpha-synuclein fibrils. In all cases, the sizes of the grid boxes were kept the same at 40 Å × 40 Å × 40 Å.

#### 2.1.4. Protein Ligand Interaction Profiler (PLIP) Analysis

To determine the binding interactions of the conjugates and the peptides with α-Syn fibrils, we conducted PLIP analysis. The most optimum binding affinity pose (lowest RMSD) of α-Syn fibrils complexed with each ligand obtained from docking studies was downloaded and uploaded as a .pdb file to the webserver PLIP (https://plip-tool.biotec.tu-dresden.de/plip-web/plip/index, accessed on 24 December 2024) [48] in order to determine the specific interactions between the respective conjugate and α-Syn fibrils. This web server provides information about non-covalent interactions between the ligands and the protein including hydrogen bonds, hydrophobic interactions, pi-stacking, and salt bridges. The resulting figures were visualized on PyMOL.

#### 2.1.5. Molecular Dynamics Simulations

To assess the stability of the α-Syn fibril–conjugate complexes, we performed molecular dynamics (MD) simulations using DESMOND within Schrödinger’s Maestro software (v. 2023-2) [49]. We utilized the OPLS 2005 force field for all simulations. The preparation for each MD simulation involved a two-step process. First, each output .pdbqt file generated from AutoDock Vina v.1.2.0 was imported into PyMOL and subsequently exported as a .mae file, which was then loaded into Maestro. Within Maestro, we utilized the Protein Preparation Wizard to enhance the structural integrity of the protein. This step involved ensuring the protein had complete side chains, adding hydrogen atoms, and forming disulfide bonds between sulfur atoms in close proximity. We also optimized the hydrogen bonding network, adjusted the orientation of hydrogen atoms, histidine rings and the terminal amide side chains of asparagine and glutamine to their correct configurations. Next, the ligand was incorporated with the receptor in the workspace, where we defined an orthorhombic simulation box with dimensions of 10 Å × 10 Å × 10 Å around the receptor. This box was set within the predefined SPC solvent model to mimic aqueous conditions. To maintain the system’s neutrality, counter ions (Na^+^ and Cl^−^) were added. We set the simulation to run for 100 nanoseconds, yielding 1000 frames for analysis. After the simulations concluded, the resulting .out.cms file was opened using the Simulation Interaction Diagram Panel in Maestro for retrieving the output data files To ensure consistency and reliability in our findings, we conducted three separate runs of each system, maintaining the same conditions throughout.

### 2.2. Laboratory Methods

#### 2.2.1. Materials

Microglial cells (SIM-A9, CRL 3265) (Mus musculus), DMEM:F12 medium (30-2006) and fetal bovine serum (FBS) were purchased from ATCC (Manasses, VA, USA). Heat-inactivated horse serum was purchased from Thermofisher Scientific. Alpha-synuclein antibody (Alexa Fluor 488) was purchased from Santacruz Biotechnology (Dallas, TX, USA). WST-8 cell proliferation assay kit, 18β-Glycyrrhetinic acid (GH) and β-Boswellic acid (BA) were purchased from Cayman Chemicals (Ann Arbor, MI, USA). The peptide sequences YYIVS, GSGGL and MPDAHL were custom ordered from GenScript. *N*-hydroxy succinimide, EDAC hydrochloride (*N*-(3-Dimethylaminopropyl)-*N*′-ethylcarbodiimide hydrochloride), solvents such as dimethyl formamide (DMF) and buffer solutions were purchased from Sigma Aldrich (Allentown, PA, USA). Interleukin-6 (IL-6) ELISA kit was purchased from Sino Biological (Chesterbook, PA, USA). α-Synuclein pre-formed fibrils were purchased from r-Peptide Inc (Watkinsville, GA, USA).

#### 2.2.2. Synthesis of Conjugates

In order to synthesize the conjugates, BA and GH each were coupled separately with the three peptides YYIVS, GSGGL and MDPAHL. The coupling was carried out using previously established peptide conjugation methods where the free carboxylic acid group of BA or GH was coupled to the N-terminal of the peptides [50]. In general, BA or GH (0.1 M) was dissolved in DMF. To activate the carboxyl groups of BA or GH, EDAC (2 mM) and NHS (8 mM) were added to the solution. The reaction mixtures were shaken at 100 rpm for one hour at a temperature of 10 °C. Following the activation of the carboxyl groups, the specific peptides were added to the reaction mixture. The reaction mixture was then shaken for 24–48 h to ensure conjugation at 10 °C. After the conjugation was complete, the product was subjected to rotary evaporation to remove the solvent, followed by recrystallization with a mixture of methanol and acetone in a 1:1 ratio. The final products obtained were further dried using a speed vacuum concentrator. The formation of the resulting conjugates was then confirmed through proton nuclear magnetic resonance (^1^H NMR) spectroscopy and FTIR spectroscopy. NMR analysis was carried out using a Bruker 400 MHz NMR spectrometer, in DMSO-d6 containing 0.3% tetramethylsilane (TMS) as the solvent.

Boswellate-peptide conjugates: *BA-GSGGL*: ^1^H NMR (400 MHz, DMSO) δ 0.82 (6H, d); δ 0.88 (9H, s); δ 0.96 (6H, d); δ 1.0 (3H, s); δ 1.1 (1H, t); δ 1.20 (2H, t); δ 1.28 (2H, t); δ 1.30 (3H, s); δ 1.32 (1H, s); δ 1.42 (1H, m); δ 1.51 (2H, m); δ 1.58 (6H, t); δ 1.65 (2H, m); δ 1.75 (4H, m); δ 1.80 (1H, d); δ 2.25 (2H, t); δ 3.82 (1H, t); δ 4.10 (6H, s); δ 4.15 (2H, d); δ 4.25 (1H, s); δ 4.62 (1H, s); δ 4.71 (1H, t); δ 5.9 (1H, s); δ 5.25 (1H, t). δ 8.1 (2H, s); δ 8.8 (3H, s).
*BA-MPDAHL*: ^1^H NMR (400 MHz, DMSO) δ 0.81 (6H, d); δ 0.85 (6H, s); δ 0.94 (6H, d); δ 1.0 (3H, s); δ 1.1 (1H, s); δ 1.20 (5H, m); δ 1.32 (3H, t); δ 1.45 (6H, m); δ 1.53 (6H, t); δ 1.60 (4H, m); δ 1.71 (4H, m); δ 1.85 (1H, d); δ 1.94 (4H, m); δ 2.1 (1H, s); δ 2.21 (2H, d); δ 2.30 (2H, t); δ 2.52 (2H, t); δ 3.0 (2H, d); δ 3.2 (2H, d); δ 3.47 (2H, t); δ 3.63 (1H, t); δ 4.32 (2H, m); δ 4.48 (1H, t); δ 4.60 (1H, q); δ 4.75 (2H, t); δ 4.88 (1H, t); δ 5. 2 (1H, s); δ 6.4 (1H, s); δ 7.5 (1H, s); δ 8.5 (5H, s); δ 8.9 (1H, s); δ 12.9 (1H, s).
*BA-YYIVS*: ^1^H NMR (400 MHz, DMSO) δ 0.82 (3H, s); δ 0.87 (6H, d); δ 0.90 (6H, s); δ 0.94 (9H, m); δ 1.0 (3H, d); 1.1 (4H, m); δ 1.21 (2H, t); δ 1.28 (3H, d); δ 1.32 (3H, t); δ 1.44 (2H, m); δ 1.50 (2H, m); (δ 1.52 (6H, m); δ 1.61 (4H, m); δ 1.68 (2H, m); δ 1.75 (1H, s); δ 2.21 (2H, d); δ 2. 41 (1H, d); δ 2.9 (1H, m); δ 3.32 (4H, s); δ 3.6 (1H, t); δ 4.01 (1H, d); δ 4.10 (2H, d); δ 4.4 (2H, m); δ 5.05 (1H, s); δ 5.22 (1H, s); δ 5.3 (1H, t); δ 6.51 (1H, s); δ 6.92 (4H, d); δ 7.1 (4H, d); δ 8.51 (5H, s); δ 9.0 (1H, s).
Glycyrrhetinate-peptide conjugates: *GH-GSGGL*: ^1^H NMR (400 MHz, DMSO) δ 0.85 (6H, d); δ 0.88 (6H, s); δ 0.92 (6H, d); δ 0.96 (1H, s) δ 1.10 (3H, s); δ 1.21 (3H, s); 1.25 (2H, t); 1.30 (3H, s); δ 1.35 (2H, t); δ 1.45 (1H, m); δ 1.58 (6H, t); δ 1.64 (2H, m); δ 1.72 (4H, d); δ 1.85 (1H, s); δ 1.95 (4H, t); δ 3.25 (1H, t); δ 3.92 (6H, s); δ 4.11 (2H, d); δ 4.44 (2H, t); δ 5.1 (1H, s); δ 5.65 (1H, s); δ 8.5 (2H, s); δ 9.2 (3H, s).
*GH-MPDAHL*: ^1^H NMR (400 MHz, DMSO) δ 0.83 (6H, d); δ 0.88 (6H, s); δ 0.92 (6H, d); 0.96 (1H, t); δ 1.1 (3H, s); δ 1.22 (5H, m); δ 1.28 (3H, s); δ 1.35 (2H, t); δ 1.45 (3H, d); 1.50 (1H, m); δ 1.58 (6H, t); δ 1.65 (2H, m); δ 1.70 (2H, m); δ 1.77 (4H, d); δ 1.94 (3H, m); δ 2.0 (2H, m); δ 2.1 (1H, s); δ 2.38 (2H, t); δ 2.64 (2H, t); δ 3.1 (2H, d); δ 3.21 (2H, d); δ 3.55 (2H, t); δ 4. 35 (1H, t); δ 4.47 (1H, t); δ 4.55 (1H, t); δ 4.71 (1H, m); δ 4.90 (1H, t); δ 4.96 (1H, d); δ 7.5 (1H, s); δ 8.42 (5H, s); δ 8.82 (1H, s); δ 12.82 (1H, s).
*GH-YYIVS*: ^1^H NMR (400 MHz, DMSO) δ 0.90 (6H, d); δ 0.95 (6H, s); δ 0.98 (1H, t); δ 1.01 (6H, d); δ 1.1 (3H, d); δ 1.18 (3H, s); δ 1.25 (4H, m); δ 1.29 (3H, s); δ 1.33 (2H, t); δ 1.52 (8H, m); δ 1.65 (2H, q); δ 1.70 (2H, m); δ 1.82 (2H, d); δ 1.95 (4H, m); δ 2.3 (1H, m); δ 2.92 (1H, m); δ 3.25 (1H, t); δ 3.40 (4H, d); δ 3.72 (2H, d); δ 3.92 (1H, t); δ 4.25 (2H, d); δ 4.88 (2H, t); δ 4.98 (1H, s); δ 5.1 (1H, s); δ 5.5 (1H, s); δ 6.83 (4H, d); δ 7.1 (4H, d); δ 8.5 (5H, s); δ 9.2 (2H, s).

#### 2.2.3. Self-Assembly of Conjugates

Each of the synthesized conjugates were allowed to self-assemble in aqueous solutions containing 2% dimethyl formamide (DMF). In general, 0.05 M solutions of each conjugate was prepared and allowed to grow over a period of one week at 25 °C. The growth of the assemblies was monitored using dynamic light scattering analysis (DLS). The Zeta Potential of the formed assemblies over time was also measured using the Zeta Sizer Nano ZS (Malvern Panalytical, Westborough, MA, USA) instrument. Samples were diluted at a ratio of 1:5 in deionized water and utilized for DLS and Zeta potential analysis. All measurements were carried out in triplicate. In general, for data analysis, the non-negatively constrained least-squares (NNLS) method was utilized as per the software provided by Malvern Instruments. The polydispersity index (PDI) which provides information about the size distribution and the extent of dispersity was in the range of 0 to 1, with lower values indicative of monodispersity and values of 0.8 and higher considered polydisperse [51]. The formed assemblies were then centrifuged, washed thrice and dried overnight in a speed-vacuum concentrator. Samples obtained were stored at 4 °C for further analysis.

#### 2.2.4. Scanning Electron Microscopy (SEM)

To examine the morphologies of the formed nanoassemblies, samples were subjected to SEM analyses using a Zeiss EVO MA10 model. Samples were dried onto carbon double-stick conducting tapes and were examined at a range of 10 kV to 15 kV at varying magnifications. The instrument was operated in EP mode for all analyses.

#### 2.2.5. Fourier Transform Infrared (FTIR) Spectroscopy

FTIR spectroscopy was conducted to confirm the structures of the nanoassemblies as well as to assess the binding interactions of each of the nanoassemblies with α-Syn fibrils. Samples were incubated in a 1:1 ratio of nanoassemblies and α-Syn fibrils and shaken at 200 rpm for 24 h. Then, samples were centrifuged and washed twice in DI water and dried using a speed vac before analysis. A Thermo Scientific, Nicolet IS50 FTIR (Thermo Scientific, Waltham, MA, USA) with OMNIC Software (9.13) (Thermo Scientific, Waltham, MA, USA) was used. In all cases, KBr pellets of samples were prepared and all spectra were taken at 4 cm^−1^ resolution with 100 scans for averaging. The sample measurements were taken between 400 and 4000 cm^−1^.

#### 2.2.6. Circular Dichroism (CD) Spectroscopy

To further confirm the binding interactions and changes in conformation in α-Syn fibrils upon association with the nanoassemblies, CD spectroscopy was conducted using a Jasco J-1500 CD Spectrometer over a wavelength range of 190 nm to 280 nm. In general, samples were prepared by incubating 4 µM of each of the nanoassemblies with 5 µg/mL preformed alpha-synuclein fibrils in clear quartz cuvettes in PBS buffer. Control samples included the neat nanoassemblies as well as untreated fibrils which were prepared at the same concentrations separately. CD spectra were recorded using at regular intervals for 48 h. The measurements were taken at room temperature with a 1 mm path length and a bandwidth of 1 nm. Baseline spectra of the control samples were subtracted from the experimental spectra to remove background noise. The spectra of the neat nanoassemblies were subtracted for each sample containing nanoassemblies associated with α-Syn fibrils. All the spectra were smoothed and converted to the mean residue ellipticity [*θ*] in deg ∗ cm^2^/dmol. To determine the secondary structural components of each of the samples containing α-Syn associated with nanoassemblies, the BeStSel (beta structure selection) web server was utilized. Each of the spectral data obtained as .txt files were uploaded to the webserver for analysis. In general, BeStSel predicts the structural elements of samples based on the reference CD spectra of known protein structures available in the server. Ref. [52] is useful for predicting the secondary structures of proteins.

#### 2.2.7. Cell Studies

##### Cell Viability

Microglial cells (SIM-A9) derived from cerebral cortex tissues collected and pooled from mouse pups were purchased from ATCC and utilized for all cell studies described in this study. Microglial cells play an important role in ingesting α-Syn [53] and in a variety of additional neural signaling pathways. To examine the impact of the nanoassemblies on the cells, viability studies were carried out using the WST-8 cell proliferation assay [54]. Cells were first grown to confluence in DMEM:F12 media supplemented with 10% FBS and 5% heat-inactivated horse serum. The media was changed twice a week to ensure proper growth of the cells. To conduct WST-8 assays, the cells were plated in triplicate at a density of 1 × 10^5^ cells/well in 96-well plates under humidified conditions at 37 °C and 5% CO_2_. After 24 h, the nanoassemblies were added at a concentration of 2 µM, 10 µM and 25 µM and allowed to incubate for 24 h. Then, as per instructions given in the WST-8 kit, the WST-8 reagent was freshly prepared. To each well, 10 µL of the WST-8 reagent was then added and incubated for 3 h at 37 °C in 5% CO_2_ incubator. Before reading the plate using a Biotek Eon plate reader, the contents of the wells were gently shaken to ensure a homogeneous distribution. The absorbance was then measured at a wavelength of 450 nm. The results reported are those obtained from the average of three separate runs for each sample. Statistical analysis was carried out using Student’s *T*-test method with n = 3.

##### Imaging

To examine the morphology of the microglial cells in the presence of the nanoassemblies, samples were plated in 24-well plates and incubated with 10 μM of each of the nanoassemblies for 24–48 h at 37 °C in a 5% CO_2_ incubator. After 24 h, cells were imaged using a BioTek Cytation C10 confocal imaging reader (Agilent Technologies, Santa Clara, CA, USA) equipped with spinning-disk confocal module and a Hamamatsu scientific (sCMOS) camera in phase contrast mode.

##### Flow Cytometry Analysis

Flow cytometry analysis was conducted to examine the impact of the nanoassemblies on the uptake of α-Syn in microglial cells. To do so, first, the α-Syn fibrils were labeled with alpha-synuclein mouse monoclonal IgG1 antibody (Alexa Fluor 488). Several studies have suggested that antibody labeling has been commonly used for flow cytometric analysis as it reduces background fluorescence uptake and generally aids in attaining optimum staining [55]. To stain the α-Syn fibrils, 4 mL of α-Syn fibril solution (25 µM) was thawed and allowed to incubate with the antibody solution (10 µM) at 4 °C for 6 h in the dark with gentle shaking. The mixture was then centrifuged to remove any unbound antibody and allowed to sit overnight at 4 °C. Separately, microglial cells that were grown to confluence were plated in triplicate in 24-well plates at a density of 1 × 10^6^ cells/well and allowed to spread in the wells overnight at 37 °C in a 5% CO_2_ incubator. Then, the tagged α-Syn fibrils were allowed to stand at room temperature for an hour in the dark followed by the addition of 25 µL of tagged α-Syn fibrils to each well, and incubated with the cells for 4 h in the dark at 37 °C in a 5% CO_2_ incubator. Then, each of the conjugate nanoassemblies (10 µM) was added to each well, and shaken gently. The well plates were then returned to the incubator. After 24 h of incubation, the medium was removed and the treated cells as well as control cells from each sample were washed with 1X PBS. The cells were then trypsinized and allowed to incubate for three minutes at 37 °C, 5% CO_2_ incubator followed by the addition of 1 mL of media to neutralize the trypsin. The contents of each well were then transferred to separate eppendorf tubes and spun at 500× *g* for 5 min. The supernatant was then removed, and the pellet was re-suspended in FACS buffer (BD Biosciences). Each sample was then transferred to a FACS tube, after being filtered through filter caps. Each sample was immediately analyzed using a BD FACSMelody flow cytometer. In general, each sample was subjected to 10,000 events during each run. The data obtained were analyzed using the software FlowJo v.10.8.1. Samples were gated first on an FSC-A/SSC-A scatter plot to exclude dead cells. This population was subsequently gated for singlets on the FSC-A/FSC-W plot. The population of live, single cells was then analyzed for COMP-FITC-A. The excitation wavelength was set to 488 nm. The data were plotted as histograms.

##### ELISA

It is well known that microglial cells play an important role not only in the internalization and processing of α-Syn, but are also involved in the generation of pro-inflammatory cytokines such as interleukin-6 (IL-6) when activated [56]. We conducted ELISA assays to measure the IL-6 expression in microglial cells in the presence and absence of nanoassemblies and α-Syn. Cells were plated at a density of 1 × 10^5^ cells per well and allowed to spread in 24-well plates for 24 h. Then, one set of the cells were allowed to incubate with 25 μL of α-Syn fibrils overnight, while the same amount of water was added to another set. Samples were left in the incubator for eight hours followed by the addition of each of the nanoassemblies (10 μM) each for 24 h at 37 °C in a 5% CO_2_ incubator to both sets. On the day of the assay, cells were trypsinized and washed with media and 1X PBS and then lysed with a Fisherbrand Model 5050 sonic dismembrator at 55 J. Then, 100 μL of the cell lysate sample or standard was added to each well of the assay plate which was pre-coated with a monoclonal antibody specific for IL-6. Prior to the assay, wash buffer was added to each well, and allowed to sit for three minutes and then aspirated followed by addition of the working solution. Preceding the assay, standard solutions, dilution buffer, wash buffer, substrate solutions and color reagents were prepared as per the manufacturer’s protocol. Standard solutions were subjected to six two-fold serial dilutions using the dilution buffer. Then, samples or standards were added to the designated wells and allowed to incubate for two hours at room temperature. Then, 300 μL of wash buffer was added and allowed to sit for 2 min followed by removal of the wash buffer. This process was repeated two more times, followed by the addition of 100 μL of the detection antibody working solution to each well, and mixed slowly and incubated for an hour and then subjected to washing again. Finally, the substrate TMB (3,3′ 5,5′-tetramethylbenzidine) solution (100 μL) was added to each well and allowed to sit in the dark at room temperature for 20 min, followed by the addition of the stop solution. Then, the plate was gently shaken and the absorbance was read at 450 nm wavelength immediately using a BioTek Eon microplate reader. The results obtained for the standard curve provided concentrations in the range of 0 pg/mL to 225.2 pg/mL. The results obtained for the samples were then tabulated.

## 3. Results and Discussion

**Figure 2:**
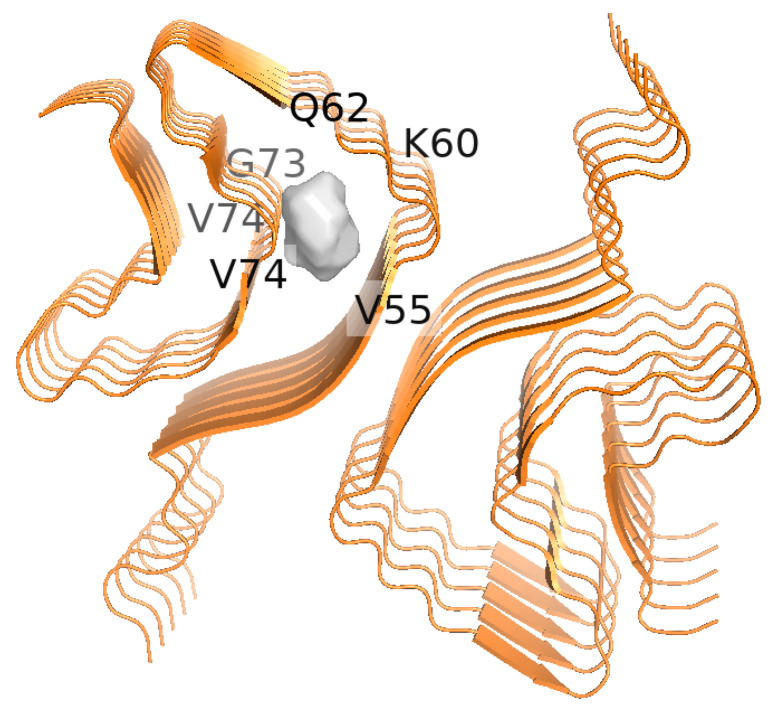
Binding pocket region of alpha-synuclein fibrils as determined by POCASA.

The BA and GH peptide conjugates were designed using ChemDraw where the carboxylic acid groups of BA and GH (C24 of BA) and (C30 of GH) were conjugated with the N-terminal of the peptides. To determine if the designed conjugates were capable of binding to α-Syn, first, binding pocket analysis of α-Syn fibrils was conducted using POCASA in order to determine the binding region of the α-Syn fibrils. As a proof of concept, the PDB ID 6H6B which represents pathogenic α-Syn fibrils (segment 1–121) was utilized to explore the interactions. Results showed a single binding pocket region (pocket number 34), which showed a volume of 123 and a volume depth (VD) value of 283. In general, the VD value is a sum of the predicted depth pockets points. Since a single binding pocket was predicted in the case of the pdb id 6H6B, this depth pocket represents the shortest distance from pocket point to the probe surface using POCASA, which utilizes a rolling probe sphere across the protein structure to detect pockets [57]. Figure 2 demonstrates a binding pocket region between residues V55 and V74 which includes multiple residues in the pre-NAC and NAC region [58] which are known to play a critical role in modulating α-aggregation.

### 3.1. Binding Affinity Studies

To predict the binding affinities of the conjugates and the peptides with α-Syn, we carried out molecular docking studies using AutoDock Vina. The results obtained are shown in Table 1. As can be seen, both BA and GH conjugates demonstrated similar binding affinities, with the GSGGL conjugates showing relatively higher binding affinity (−10.2 kcal/mol). Moreover, the conjugates showed higher binding affinities compared to the unbound neat peptides. Furthermore, we also examined the binding affinities of the conjugates with four additional amyloidogenic proteins known to misfold and lead to neural degeneration or are seen in the case of Type II diabetes (in the case of the islet amyloid fibrils) (Supplementary Information Table S1). As can be seen, in all cases, the conjugates were predicted to demonstrate a relatively higher binding affinity toward α-Synuclein compared to the other amyloidogenic peptides. These results indicate that while the conjugates may not be predicted to show exclusive binding interactions with α-Synuclein fibrils, they show preferential binding toward α-Syn. Among the amyloidogenic proteins, the conjugates showed relatively moderate binding with amyloid beta fibril (Type B), with GH-Y specifically showing a relatively high binding affinity (−9.2 kcal/mol), though the value is lower than that observed for α-Synuclein fibrils at −10.2 kcal/ mol with GH-Y. These results indicate that the conjugates may be potentially utilized to study the impact on the misfolding and aggregation of other dementia-related proteins. This is particularly of importance given that studies have recognized the interactions between beta-amyloid peptides involved in Alzheimer’s disease and alpha-synuclein that may lead to further aggravation of neuropathological diseases [59].

#### PLIP Analysis

To further determine the regions of alpha-synuclein involved in binding with the conjugates and the peptides, we conduced PLIP analysis. As can be seen in Table 2, for the conjugates, interactions occurred in the predicted binding pocket region encompassing the pre-NAC and the NAC regions. Interactions that were commonly seen across all conjugates and peptides were those with Thr54, Ala56, Glu61 and Gly73-Thr75. Specifically, the GH-Y conjugate showed the most interactions mainly with residues within the NAC region through a total of eleven hydrogen bonds and six hydrophobic interactions. In comparison, GH-G interacted with α-Syn fibrils through ten hydrogen bonds (four of which were formed with Thr75 and three with Thr59) and four hydrophobic interactions while GH-M formed nine hydrogen bonds with four of the hydrogen bonds formed with Thr59 and two each with Val74 and Gly73. In addition, two hydrophobic interactions were seen with Thr54 and Ala56.

Among the BA–peptide conjugates, the BA-Y conjugate and BA-M conjugate formed ten hydrogen bonds respectively, while BA-G formed eight hydrogen bonds. Interestingly, the number of hydrophobic interactions were higher for the BA–peptide conjugates compared to the GH–peptide conjugates. This may be attributed to the structural differences between GH and BA. Specifically, BA-Y formed eight hydrophobic interactions (three with Thr54 and two with Thr59) while BA-G formed eight hydrophobic interactions, five of which were with Thr59. The BA-M conjugate also formed eight hydrophobic interactions, primarily with Thr54, Ala56 and Thr59 showing two interactions each, while Glu61 and Thr75 of the NAC region formed one hydrophobic interaction each with BA-M. The Thr75 residue also interacted through a single hydrophobic interaction with BA-Y, with which it also formed four hydrogen bonds. Interestingly, the BA-G conjugate only formed a single hydrogen bond with Thr75, and all of the hydrophobic interactions occurred with pre-NAC region residues. These results are promising given that amino acid residues in the range of 71–82 which encompass the central hydrophobic region of ASyn are considered crucial for aggregation and misfolding of α-Syn [60,61]. Furthermore, the known ASyn aggregation inhibitor synuclean-D (2-hydroxy-5nitro-6-(3-nitrophenyl)-4-(trifluoromethyl) nicotinonitrile) was also found to bind to residues such as Val55, Thr59, Gly73, Ala53 and Thr72 [62].

Among the neat peptides, the peptide GSGGL showed the highest number of hydrogen bond interactions (eleven) with residues such as Thr59, Glu61, Gly73-Thr75, while only showing three hydrophobic interactions. MPDAHL on the other hand showed only one hydrophobic interaction with Thr54 and seven hydrogen bond interactions with the same residues as those seen for GSGGL. In comparison, the peptide YYIVS showed a relatively higher number of hydrophobic interactions (six), including multiple interactions with Thr54 and Thr59, and a single interaction with Ala56. In addition, YYIVS formed eight hydrogen bonds with three hydrogen bonds formed with Gly73 and two each with Thr75 and Glu61. The higher numbers of hydrophobic interactions seen for the YYIVS peptide can be attributed to the presence of hydrophobic residues such as tyrosine, isoleucine and valine and only one hydrophilic residue (serine). Additionally, the bond distances were generally found to be relatively higher in the case of hydrophobic interactions compared to hydrogen bonds, indicative that the hydrogen bonds formed were tighter and may play an important role in stabilizing these complexes.

### 3.2. Molecular Dynamics (MD) Simulations

**Figure 3:**
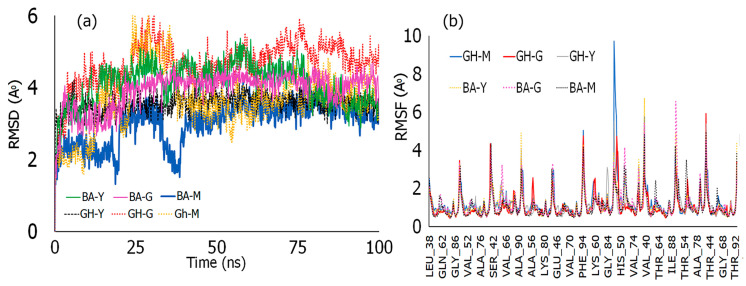
(**a**) Root mean square deviations and (**b**) root mean square fluctuations of BA–peptide and GH–peptide conjugates over 100 ns simulations. (Y, G and M represent the peptide components of the conjugates: YYIVS, GSGGL and MPDAHL, respectively).

MD simulations were conducted for the conjugates with α-Syn fibrils to shed further light into the interactions of the conjugates with α-Syn and to explore the stability of the complexes formed. As can be seen in Figure 3, the root mean square deviation (RMSD) values (Figure 3a) for the BA–peptide conjugates were found to be within 4.0 Å indicative of the formation of stable complexes [63]. Overall, BA-G and BA-Y showed very little deviations over the course of the simulation, while BA-M showed deviations for the first 40 ns and stabilized at 2.8 Å after 50 ns. Amongst the GH–peptide conjugates, the most stable complex was seen for GH-Y, which displayed an RMSD value of ~3.5 Å for the entire simulation. Both GH-M and GH-G conjugates on the other hand showed relatively higher RMSD values, particularly within the first 40 ns of the simulation. After 40 ns, however, the GH-M complex showed a more stable pattern, and values were found to be within 4.0 Å. However, the GH-G conjugate showed deviations between 4.8 Å and 5.6 Å throughout the simulation likely because of the movement of the conjugate within the multiple chains of α-Syn fibrils. As with the conjugates, each of the peptides also showed RMSD values within 4.0 Å (Supplementary Information Figure S1) in all cases demonstrating stable binding.

**Figure 4:**
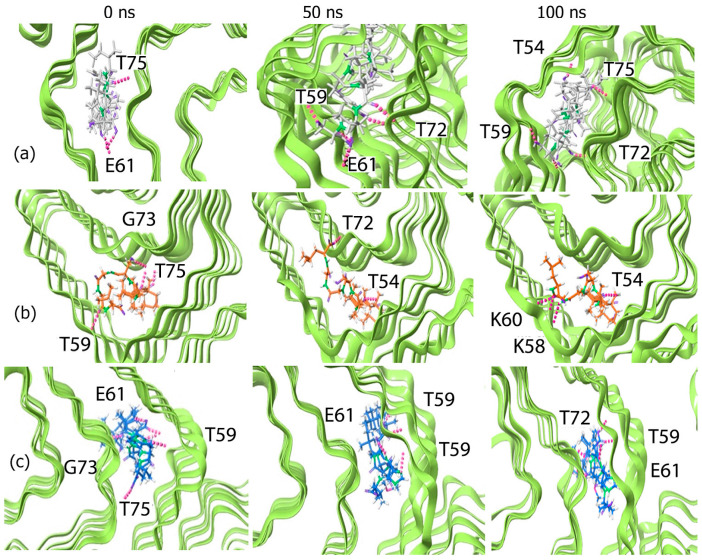
Trajectory snapshots of GH–peptide conjugates complexed with α-Syn fibrils at 0 ns, 50 ns and at 100 ns over a 100 ns simulation. (**a**) GH-YYIVS, (**b**) GH-GSGGL and (**c**) GH-MPDAHL.

Subsequently, the root mean square fluctuations of the protein backbone of α-Syn was examined upon the formation of complexes with each of the conjugates. The results (Figure 4b) indicated that in general, the interactions occurred at similar residues in all cases including those with Leu38, Lys58, Lys60, Val95, Glu83 and Thr81. The highest RMSF value was seen for GH-M at Leu38 though all conjugates showed fluctuations in this region. This is promising given that the region between residues (36–42) belongs to the P1 region of α-Syn and is known to modulate amyloid formation, and is therefore a therapeutic target [64]. In addition, complexation with BA-Y and GH-G also resulted in higher RMSF values (6.7 Å and 5.9 Å, respectively) at Val95 across multiple chains which may indicate that upon binding with those conjugates, the aggregation of fibrils may be disrupted [65]. Additionally, GH-Y also showed a relatively higher RMSF value (3.1 Å) at Glu83 compared to all other conjugates, while GH-G showed a relatively higher RMSF value (2.6 Å) at Thr59 compared to other conjugates. These results also corroborate with the PLIP analysis that the conjugates interacted with multiple residues of the pre-NAC and NAC domain.

To visualize the interactions occurring during the course of the simulation, we examined the trajectory snapshots taken at different time points of the simulation. In the case of the GH–peptide conjugates, (Figure 4) the conjugates appear to spread out and extend into the α-Syn fibrils as the simulation progresses, making contact with multiple chains. The change is particularly evident in the case of the GH-Y conjugate where initially the conjugate appears compact within the binding cavity; however, as the simulation progressed the conjugate became more deeply embedded within the fibrils.

**Figure 5:**
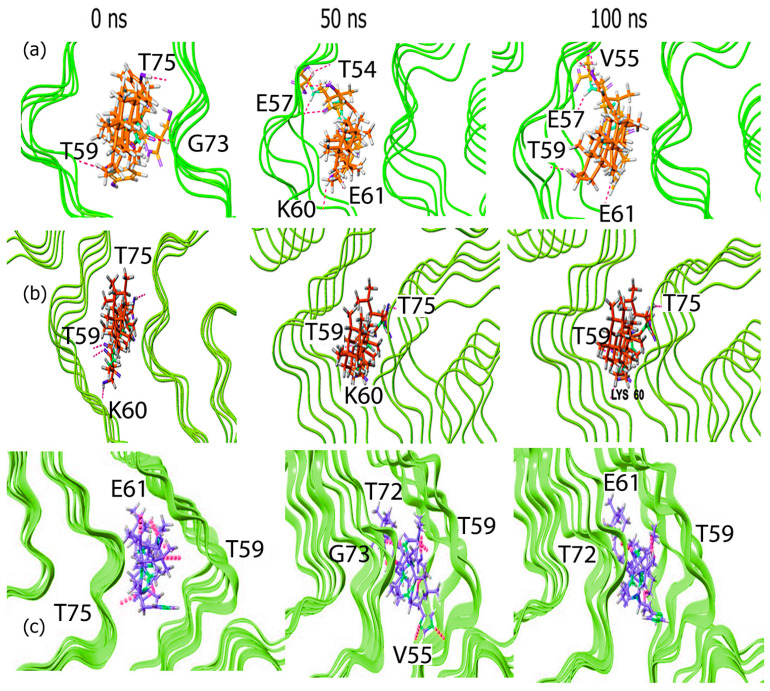
Trajectory snapshots of BA–peptide conjugates complexed with α-Syn fibrils at 0 ns, 50 ns and at 100 ns over a 100 ns simulation. (**a**) BA-YYIVS, (**b**) BA-GSGGL and (**c**) BA-MPDAHL.

In the case of the BA–peptide conjugates (Figure 5), relatively less changes were observed during the course of the simulation for the BA-Y conjugate upon complexing with α-Syn, which explains the low and stable RMSD values seen. For the BA-M conjugate, however, there was an increase in interactions over time, with the conjugate binding to multiple chains of α-Syn and moving deeper into the α-Syn cavity by the end of the simulation. A similar result was seen for the BA-G conjugate, though to a lesser extent compared to the BA-M conjugate. All of the conjugates bound to similar regions encompassing the binding pocket region that was predicted by POCASA.

Overall, based on the in silico studies, we found that the designed conjugates interacted with fibrillar α-Syn and formed stable complexes. Specifically, demonstrating interactions with critical regions of multiple chains of α-Syn. To further validate the results, laboratory studies were conducted.

### 3.3. Laboratory Studies

Each of the conjugates were synthesized and the products obtained were self-assembled. In general, nanoassemblies containing bioactive functionalities can be fine-tuned to form a multitude of structures and incur several advantages including a high surface to volume ratio, better targeting ability and potentially higher cellular uptake [66,67].

#### 3.3.1. Formation of Nanoassemblies

**Figure 6:**
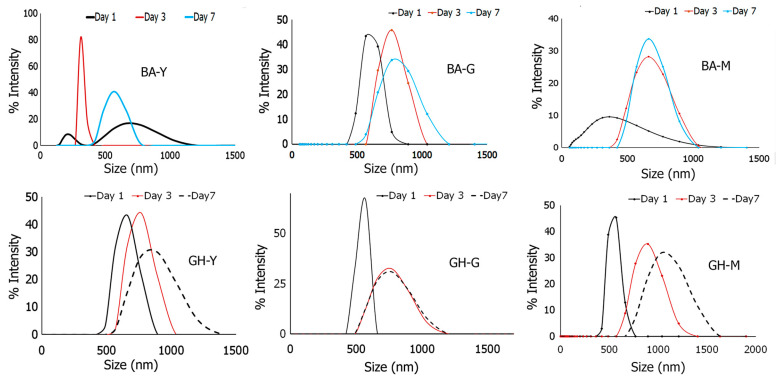
Comparison of Dynamic Light Scattering Analysis for each of the nanoassemblies formed over a period of one week.

Each of the conjugates were synthesized and self-assembled under aqueous conditions. Samples were grown for seven days and the growth was monitored using dynamic light scattering analysis. As can be seen in (Figure 6), the assemblies were found to increase in diameter over time and were relatively polydisperse. On average, the diameter of the Boswellate–peptide nanoassemblies were found to be between 500 nm and 800 nm after seven days of growth. Specifically, the average diameter of BA-Y conjugate nanoassemblies was found to be 570 ± 40 nm, while that of BA-M assemblies was 660 ± 25 nm with polydispersity indexes values of 0.4 and 0.8, respectively, after seven days of growth. The BA-G nanoassemblies were found to be larger in size displaying an average diameter of 760 ± 32 nm with a polydispersity index value of 0.7. Interestingly, it appears that in the case of the BA-Y nanoassemblies, the particle sizes decreased between one and three days and became monodisperse, indicative that the assemblies were likely folding up to assemble; however, after seven days, there was a slight increase in growth, likely because of the particles forming aggregates overtime. The GH–peptide assemblies appeared to form larger assemblies. Specifically, the average diameter of the GH-G nanoassemblies was found to be 760 ± 20 nm (polydispersity index value of 0.8), while that of GH-Y was found to be 890 ± 25 nm (polydispersity index of 0.6). The GH-M nanoassemblies formed structures that were in the range of 900 ± 15 nm in diameter (polydispersity index of 0.8). These results indicate that the BA–peptide conjugates may form relatively more compact assemblies compared to the GH–peptide assemblies. We also compared the zeta potential values of each of the assemblies over time. In general, each of the nanoassemblies displayed negative values. This is expected given the presence of the terminal carboxyl group of the peptide moiety of the conjugates. At the end of one week, the average zeta potential value for GH-M was −25.2 mV, while that of BA-M was −20.6 mV. On the other hand, in the case of BA-Y, the value was found to increase over time, showing a value −1.9 mV at the end of the one-week period, while those obtained for GH-Y decreased from −0.12 mV to −21.5 mV over a period of one week. This indicates that the carboxyl groups of the GH-Y nanoassemblies are more exposed to the solvent compared to the BA-Y nanoassemblies overtime. In general, these values are similar to the range of values seen for deoxycholic acid-survivin peptide-based nanoassemblies coated with lipid membranes which were shown to form core-shell peptide assemblies [68]. The values obtained for the GH-G and BA-G nanoassemblies were also found to be at −9.8 mV and −24.2 mV, respectively. Overall, higher values are considered to be more stable; thus, it appears that the MPDAHL peptide conjugates and the BA-G and GH-Y nanoassemblies demonstrated higher stability.

**Figure 7:**
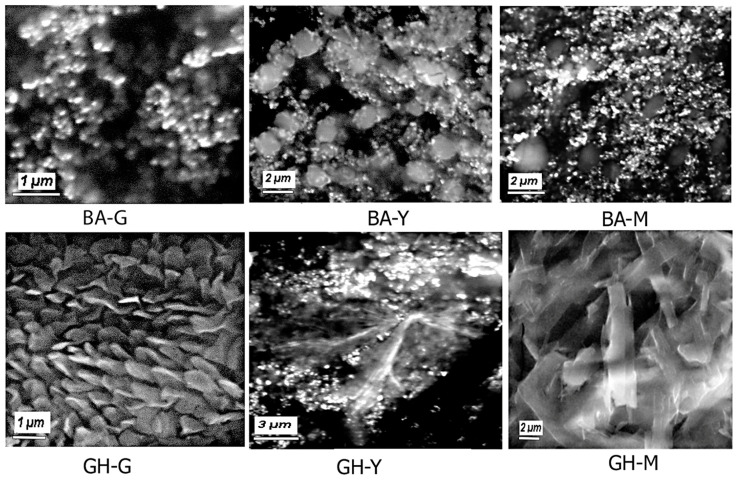
Scanning electron microscopy images of self-assembled BA–peptide and GH–peptide conjugates. BA = Boswellate; GH = glycyrrhetinate; M = MPDAHL; G = YYIVS; and G = GSGGL.

To investigate the morphologies of the assemblies, we carried out SEM analysis. The results are shown in Figure 7. Interestingly, while BA-G conjugate formed spherical nanoassemblies that appear to aggregate, the BA-Y and BA-M nanoassemblies showed a mix of vesicle-like structures and spherical nanoassemblies. The vesicle-like structures are more particularly prominent in the case of the BA-Y nanoassemblies. These results corroborate with previous studies, where Boswellic acid has been shown to self-assemble into nanospheres [69]. However, given the presence of the peptide moieities within the conjugates, there appears to be a mix of structural shapes and sizes, which is expected for peptide-based nanoassemblies given the variety of non-covalent interactions involved [70]. Interestingly, the GH–peptide nanoassemblies demonstrated varied structures based on the peptide that was conjugated. In the case of GH-G, platelet-like uniform assemblies were observed, while GH-Y showed the formation of a mix of fibrillar and spherical assemblies. GH-M on the other hand showed the formation of thicker nanofibrillar structures, which would account for the larger size ranges seen in the DLS results. While both BA and GH contain hydroxyl groups on ring A, the GH moiety also contains a keto group on ring C, which increases its polarity [71]. This difference is likely to induce a change in the self-assembly process of these conjugates. Furthermore, the YYIVS peptide contains only one hydrophilic serine residue and two tyrosine residues, in addition to a valine and a leucine residue, all of which are hydrophobic. These adjacent tyrosine residues can induce stacking interactions, while the isoleucine and valine residues can promote hydrophobic interactions [72] with the BA and GH moieties. The BA-G and GH-G conjugates on the other hand are relatively less hydrophobic as the peptide moiety only contains one hydrophobic residue (leucine), while it contains three glycine residues and one serine residue all of which promote hydrogen bond interactions that may promote the formation of fibrillar structures in addition to nanospheres. The hydrophobicity of those conjugates would be primarily attributed to the GH and BA moieties and the leucyl moiety. In addition to fibrillar structures, the GH-G conjugates also show a similar spherical morphology that was seen upon the formation of nanoemulsions of poly (lactic-co-glycolic acid)-GH nanoparticles which contains both hydrophobic and hydrophilic moieties [73]. The MPDAHL conjugates with BA and GH on the other hand contain histidine and proline moieties which can participate in stacking interactions, and histidine which is known to play a key role in molecular self-assembly processes [74] can also participate in π–cation interactions [75]. In previous studies, glycyrrhetinic acid when conjugated with the peptide sequence GFFYKE and ERGD through a spacer as well as with curcumin was found to form supramolecular hydrogels that demonstrated fibrillar morphologies. Thus, it is likely that the GH-YYIVS and GH-MPDAHL structures are forming fibrillar and tubular structures as a result of the intramolecular and intermolecular interactions of the peptides with the terpene moieties described above.

#### 3.3.2. Interactions of GH–Peptide and BA–Peptide Nanoassemblies with α-Synuclein

**Figure 8:**
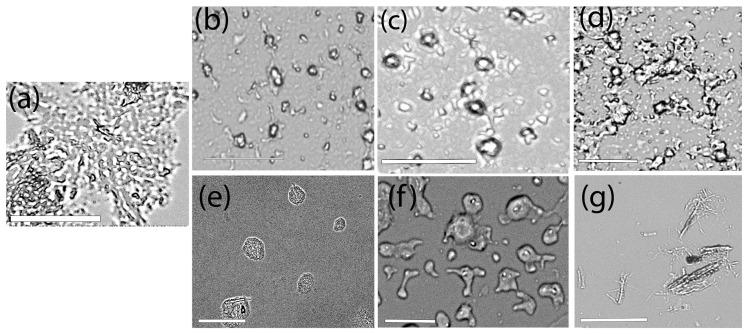
Comparison of interactions of alpha-synuclein fibrils with self-assembled nanoconjugates. (**a**) α-Syn fibrils (untreated); (**b**) BA-G nanoassemblies with α-Syn; (**c**) BA-Y assemblies with α-Syn; (**d**) BA-M nanoassemblies with α-Syn; (**e**) GH-G nanoassemblies with α-Syn; (**f**) GH-Y nanoassemblies with α-Syn; and (**g**) GH-M nanoassemblies with α-Syn. All samples were incubated with α-Syn for 48 h, centrifuged, washed and then imaged using confocal microscopy. Images were taken in brightfield mode. Scale bar = 4 μm.

The binding interactions of each of the nanoassemblies with α-Synuclein fibrils were first probed using brightfield imaging. In general, the nanoassemblies were incubated with α-Syn for 48 h, centrifuged and washed before imaging. As can be seen in Figure 8, the incorporation of α-Syn led to the formation of hybrid supramolecular assemblies of BA-Y and BA-M with α-Syn, while the BA-G nanovesicles appeared to largely attach to the α-Syn fibrils on the periphery of the nanovesicles. The BA-M nanostructures as well as BA-Y appeared to form gelatinous supramolecular hydrogel structures, with the nanospheres and nanovesicles forming a network. The GH-G assemblies interestingly appeared to bind uniformly to the fibrils efficaciously, forming multi-layered structures. The GH-Y nanoassemblies appear to undergo a change in morphology, where the α-Syn appears to form fused structures with the nanoassemblies intertwined in between the GH-G assemblies. The GH-M nanoassemblies however, appeared to maintain their fibrillar/tubular morphology, and attachment of α-Syn nanofibers was clearly seen throughout the assemblies. These results confirm the incorporation of the α-Syn fibrils with the GH–peptide and BA–peptide nanoassemblies and suggests that the chemical microenvironment of the nanoassemblies is likely altered during the binding to α-Syn fibrils. A similar phenomenon has also been observed in the case of lipid structures upon binding to α-Syn [76,77]. These results indicate that the α-Syn fibrils appear to induce a reorganization in the structures of the nanoassemblies, particularly all of the Boswellate assemblies, as well as the glycyrrhetinate assemblies GH-Y and GH-G. It is likely that the insertion of α-Syn causes the protrusion and expansion of the nanoassemblies, which is particularly more evident in the case of GH-Y, BA-Y and the BA-M structures. This type of insertion may be similar to those observed when amyloid peptides interact with lipid bilayers that lead to a lateral expansion and changes in morphologies of the bilayers [78,79]. While the exact mechanism is not known, and will require further study, it appears that the presence of the free carboxylate groups of the nanoassemblies renders the assemblies a relative negative charge (as was also observed in the zeta potential analyses); it is likely that the presence of multiple lysine residues toward the N-terminal of α-Syn and within the NAC region of α-Syn may promote binding interactions with the nanoassemblies. In addition, the presence of residues such as valine, phenylalanine, leucine and alanine may promote hydrophobic interactions with the amphiphatic nanoassemblies. Additionally, in the case of the GH-Y and GH-G nanoassemblies, a mitigation in the fibrillar structures of α-Syn is observed due to facile insertion compared to the other assemblies, which may be due to the formation of smaller, unbranched oligomers upon incorporation within the nanoassemblies. Further mechanistic studies would be necessary to determine the exact mechanism of insertion and oligomerization involved and will be reported in a separate study.

**Figure 9:**
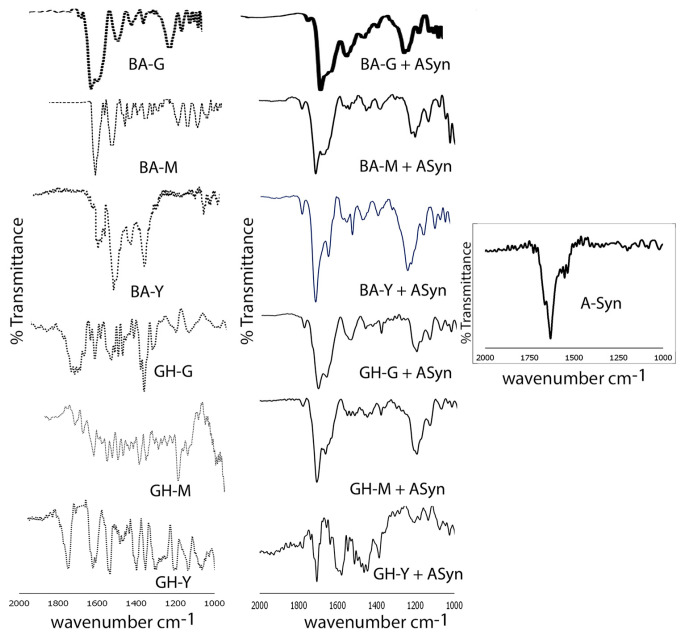
Comparison of FTIR spectra of BA–peptide and GH—–peptide nanoassemblies before and after binding with α-Syn. The spectrum of neat α-Syn is also shown to the far right of the image. M = MPDAHL; G = GSGGL; Y = YYIVS.

To further examine the interactions of α-Syn with the assemblies, we conducted FTIR spectroscopy. The results are shown in Figure 9. As can be seen, upon binding to α-Syn, key peak shifts are observed in all cases. In the case of the BA-G nanoassemblies, the amide I carbonyl peaks are seen at 1669 cm^−1^ with a shoulder at 1711 cm^−1^ while the amide II peak was observed at 1538 cm^−1^. The peak at 1711 cm^−1^ was assigned to the terminal carboxyl group of the C-terminal of the peptide moiety. The presence of the amide band also confirms the formation of the conjugate, as it was not seen for neat BA. The hydroxyl peak is seen at 3380 cm^−1^ and the C-H stretching vibrations are seen between 2834 cm^−1^ and 2946 cm^−1^. The C-O peak is seen at 1202 cm^−1^, while the peaks at 1376 cm^−1^ and 1453 cm^−1^ are representative of the C-H bending peaks. Upon binding to α-Syn, relatively broad peaks are seen at 1706 cm^−1^ and at 1640 cm^−1^, while the amide II peak appears at 1558 cm^−1^ and C-O peaks are seen at 1238 cm^−1^ and at 1201 cm^−1^. The broad amide peaks may be attributed to a mix of peptide structural elements as a result of the conjugate interacting with α-Syn including loops, turns and helices [80,81]. These shifts are likely due to changes in hydrogen bonds formed between the peptide moieties of the conjugates and the amide groups of α-Syn fibrils. Notably, the amide I peak for the BA-M nanoassembly was seen at 1686 cm^−1^. Upon binding to α-Syn, however, split peaks were observed in this region at 1667 cm^−1^ and at 1712 cm^−1^. The peaks in the C-O region were also shifted upon binding to α-Syn. Upon binding to α-Syn, the BA-Y nanoassemblies showed significant shifts, displaying the carbonyl peaks at 1636 cm^−1^ and at 1709 cm^−1^, and the C-O peak at 1213 cm^−1^ compared to unbound nanoassemblies which showed peaks at 1650 cm^−1^ and at 1677 cm^−1^, in addition to a strong C-H deformation peak that was seen at 1425 cm^−1^ along with the C-O peak at 1203 cm^−1^ for the nanoassemblies.

A similar trend was observed for the GH–peptide bound α-Syn assemblies where amide I peaks were observed at 1665 cm^−1^ and the peptide carboxyl peak was seen at 1701 cm^−1^, while the amide II peak was seen at 1555 cm^−1^ for GH-MPDAHL assemblies and the C-O peak was seen at 1201 cm^−1^. The α-Syn-bound GH-Y assemblies showed carbonyl peaks at 1706 cm^−1^ and at 1637 cm^−1^, while the amide II peak was observed at 1577 cm^−1^ and the C-O peak was seen at 1213 cm^−1^ and at 1175 cm^−1^. In comparison, the unbound GH-M nanoassemblies showed the amide I peak at 1674 cm^−1^, and the amide II peak was seen at 1536 cm^−1^. Strong C-H bending peaks were seen at 1424 cm^−1^ and at 1405 cm^−1^. In addition, the C-O peaks were seen at 1265 cm^−1^. The unbound GH–peptide assemblies also displayed an additional carbonyl peak at 1779–1770 cm^−1^ which is attributed to the carbonyl group in ring C of the GH moiety. For GH-Y, the amide I peak was at 1648 cm^−1^ with a shoulder at 1633 cm^−1^. The amide II peak was seen at 1557 cm^−1^ and the C-O peaks were seen at 1203 cm^−1^. The C-H bending peak was observed at 1415 cm^−1^ and at 1367 cm^−1^. The GH-G nanoassemblies showed peaks at 1770 cm^−1^ and 1649 cm^−1^, while the amide II peak was seen at 1556 cm^−1^. The C-H bending peak was seen at 1415 cm^−1^ and at 1376 cm^−1^ while the C-O peak was seen at 1250 cm^−1^. In contrast upon binding to α-Syn, we observed amide I peaks at 1709 cm^−1^ with a shoulder at 1667 cm^−1^, the amide II peak was observed at 1540 cm^−1^ and the C-O peak was seen at 1203 cm^−1^. These results further confirm the incorporation of α-Syn with the nanoassemblies. Additionally, when compared to the FTIR spectrum of neat α-Syn, the incorporation of α-Syn with the assemblies is further established.

The conformational changes of α-Syn upon binding with the assemblies was probed using CD spectroscopy. The results are shown in Table 3. The spectra obtained were analyzed using the BeStSel webserver in order to determine the percentages of secondary structural elements present [82]. As can be seen, upon binding with the nanoassemblies, conformation changes were induced in α-Syn. In general, the interactions resulted in the appearance of parallel β-sheet structures (which are known to have slower aggregation rates compared to antiparallel β-sheets) in the case of all of the Boswellate–peptide nanoassemblies after 24 h; however, at 48 h, those were no longer seen. All three of the Boswellate–peptide nanoassemblies resulted in increased disordered structures. Interestingly, both BA-Y and BA-M showed the appearance of helical structures at the 24 h mark; however those were not seen after 48 h. The BA-G on the other hand appears to induce lesser changes in α-Syn comparatively over time, where only a slight reduction in anti-parallel β-sheet structures is seen, with a concomitant increase in disordered structures.

In the case of the GH-Y nanoassemblies, over 48 h, we observed an increase in the appearance of helical structures, while the percentage of antiparallel β-sheets decreased, and parallel β-sheets were formed at 48 h. In addition, while the percentage of disordered structures initially increased (at 24 h), it was found to decrease by 14.1% after 48 h compared to initial conditions. Upon binding with GH-G nanoassemblies, α-Syn also showed increased formation of helices over time, accompanied by the appearance of parallel beta-sheets and a decrease in the percentage of disordered structures. In contrast, the GH-M nanoassemblies resulted in the appearance of alpha-helices at 24 h. However, after 48 h, those were no longer seen, and there was a minute change in the percentage of disordered structures, though the percentage of antiparallel beta-sheets showed an increase at 48 h. These results indicate that upon binding to the nanoassemblies, changes are induced in the α-Syn fibril conformation, and furthermore those changes are more pronounced and dynamic upon interacting with the BA–peptide assemblies compared to GH–peptide assemblies.

#### 3.3.3. Cell Studies

##### Cell Viability

**Figure 10:**
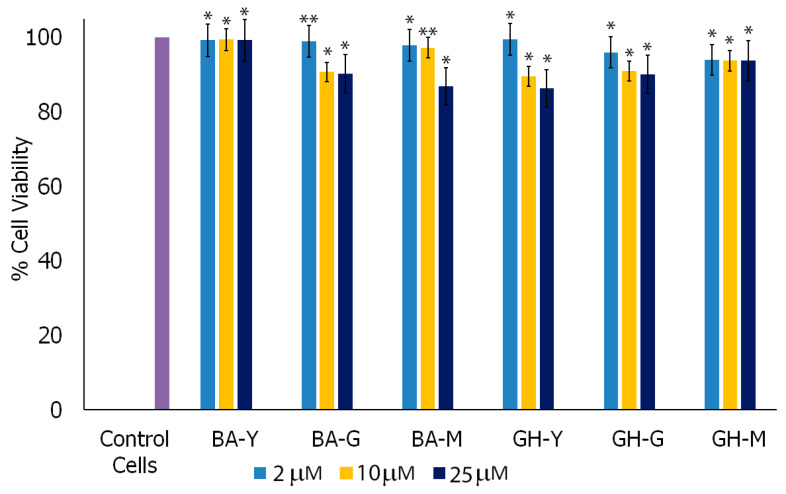
Cell viability results for microglial cells after 48 h of incubation with BA–peptide and GH–peptide assemblies. Y, G and M represent the peptide components YYIVS, GSGGL and MPDAHL, respectively, while GH and BA represent the glycyrrhetinate and Boswellate components of the conjugates, respectively. Data expressed are the mean (n = 3) with error bars indicating standard deviations. * *p* < 0.05; ** *p* < 0.01.

To examine the biocompatibility of the nanoassemblies, we conducted cell viability studies with microglial cells in the presence of each of the nanoassemblies at varying concentrations. Specifically, microglial cells were utilized as they are known to play a critical role in interacting with α-Syn and in α-Synucleinopathies [83]. The results obtained are shown in Figure 10. In general, the nanoassemblies showed high cell viability. Overall, the nanoassemblies showed viability between 88% and 98.9% depending upon the concentrations utilized. The highest viability was shown by BA-Y nanoassemblies across all concentrations, while GH-Y demonstrated the highest viability at lower concentrations (2 μM and 10 μM), although the viability was still fairly high (88%) at 25 μM concentration. These results indicated that the nanoassemblies were not cytotoxic to microglial cells.

**Figure 11:**
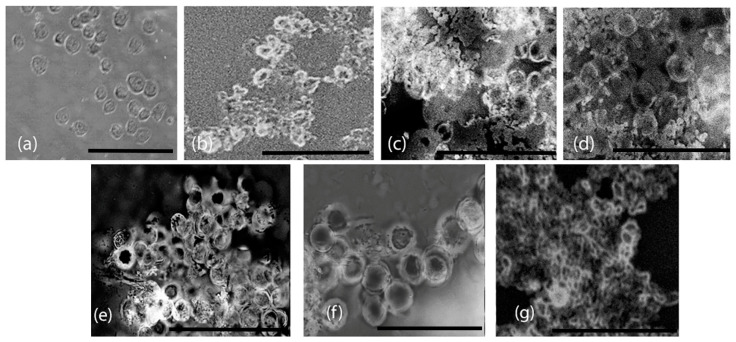
Interactions of microglial cells with nanoassemblies. (**a**) Control untreated cells; (**b**) cells incubated with BA-G assemblies; (**c**) cells incubated with BA-G assemblies; (**d**) cells incubated with BA-G assemblies; (**e**) cells incubated with BA-G assemblies; (**f**) cells incubated with BA-G assemblies; (**g**) cells incubated with BA-G assemblies. Images are taken after 24 h of incubation. Scale bar = 50 μm.

To examine the morphology of the cells upon interacting with the nanoassemblies, phase contrast optical microscopy images were taken after incubation with the assemblies. The results obtained after 24 h of incubation with the nanoassemblies are shown in Figure 11. The untreated microglial cells displayed an oval morphology. In general, the cells appeared to maintain a similar morphology after treatment with the nanoassemblies. In the case of the BA–peptide nanoassemblies, the assemblies appear to adhere to the cells, particularly around the periphery of the cells forming a scaffold-like structure in which the cells appear to be embedded. This is particularly evident in the case of the BA-Y and BA-M assemblies. A similar trend was observed for the GH-G and GH-Y assemblies, where the assemblies appeared to adhere to the cells around the edges. In the case of the GH-M assemblies, interestingly, the cells appeared to form cell–scaffold matrix-like structures as cells were embedded completely within the fibrous GH-M assemblies. These results demonstrate that the microglial cells efficiently adhered to all of the assemblies.

**Figure 12:**
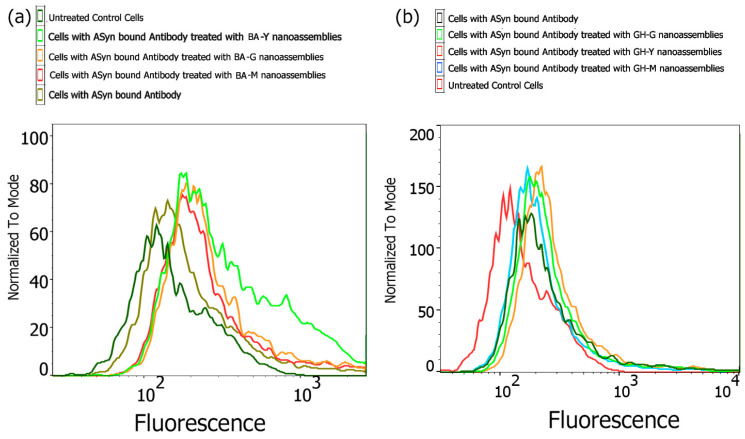
(**a**,**b**) Flow cytometry analysis showing uptake of alpha-synuclein in the presence and absence of nanoassemblies.

To examine if the nanoassemblies had an impact on the cellular uptake of α-Syn, we conducted FACS analysis. It is well known that in PD, extracellular α-Syn tends to aggregate and form neurotoxic Lewy bodies. Studies have shown that α-Syn is efficiently internalized by microglial cells and then degraded through autophagy [84]. Recent studies have also shown that microglia play a vital role in spreading of the α-Syn aggregates between cells, which may contribute to the propagation of α-Syn neuropathology in Parkinson’s disease and induce neuroinflammation [85]. The dysfunction of intracellular degradation pathways such as the autophagy–lysosomal pathway or the ubiquitin–proteasome pathway may result in further accumulation of α-Syn within neurons and microglia leading to impaired mechanisms of clearance that may further cause the formation of toxic α-Syn aggregates and accelerate neuropathological effects [86,87,88]. Thus, we examined if the nanoassemblies had an impact on the cellular uptake of α-Syn fibrils. The results are shown in Figure 12. As can be seen upon treatment with the nanoassemblies, we observed that there was an enhancement in the uptake of α-Syn. Specifically, among the BA–peptide nanoassemblies, the BA-Y nanoassemblies demonstrated the highest increase in uptake of α–Syn, while BA-G nanoassemblies and BA-M assemblies showed similar effects, with BA-G nanoassemblies demonstrating a marginally higher internalization of α-Syn compared to BA-M. Regardless, compared to the controls, all of the BA–peptide nanoassemblies appear to have enhanced the internalization of α-Syn. Similar trends were observed for the GH–peptide assemblies with the GH-Y nanoassemblies showing relatively higher enhancement in the internalization of α-Syn followed by GH-G nanoassemblies and GH-M nanoassemblies. These results are promising given that higher uptake α-Syn by microglial cells may lead to improved degradation of α-Syn. While the exact mechanism of promotion of internalization is not known, and will require further study, it is likely that the nanoassemblies bound to α-Syn are internalized through endocytosis. In previous work, it has been shown that α-Syn is internalized through endocytosis [89].

**Figure 13:**
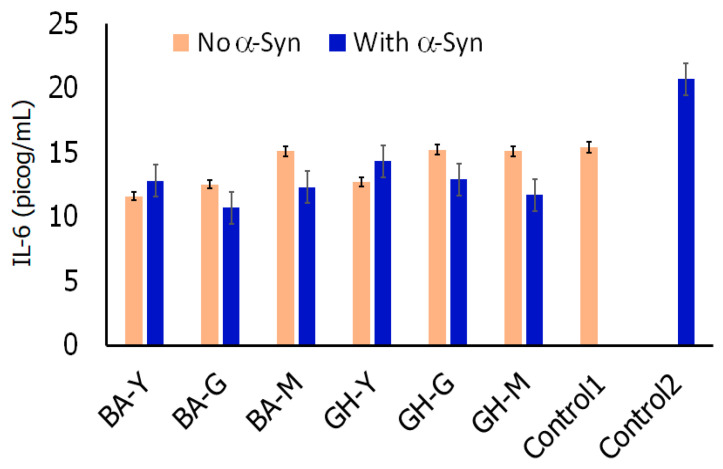
Expression of interleukin 6 in the presence and absence of nanoassemblies and α-Syn fibrils. Control 1: Microglial cells with no treatment. Control 2: Microglial cells with α-Syn fibrils, no assemblies were added.

In addition, α-Syn fibrils have been known to activate Toll-like receptor 2 (TLR2) in microglia which are known to further activate the intracellular NF-κB pathway and cause the release of pro-inflammatory cytokines such as IL-6 among others [90]. We therefore conducted ELISA assays to determine if the nanoassemblies had an impact on the release of IL-6 both in the presence and in the absence of α-Syn fibrils. The results are shown in Figure 13. Our results indicated that for cells without nanoassemblies, the IL6 released in the presence of α-Syn was higher (21.2 pg/mL) compared to those found in the absence of α-Syn (15.1 pg/mL). This corroborates with previous work which has shown that α-Syn activates the pro-inflammatory cytokines like IL-6. Interestingly, upon treatment with all of the Boswellate–peptide nanoassemblies in the presence of α-Syn, we observed a decrease in IL-6, with BA-G nanoassemblies showing a relatively higher impact with lowest IL-6 expression. The GH–peptide nanoassemblies also appeared to show a reduction in IL-6 expression, particularly upon treatment with GH-M nanoassemblies. Thus, our results indicate that the nanoassemblies are not only binding with α-Syn but are also implicated in mitigating the expression of IL-6. In the absence of α-Syn, the nanoassemblies did not show a significant effect. In previous work, Boswellic acid and its analogues containing 4-amino derivatives have been shown to reduce the expression of IL-6 [91]. Similarly, glycyrrhetinic acid has also been shown to reduce the expression of pro-inflammatory cytokines such as IL-6 as have certain antioxidant peptides [92,93]. Thus, our results indicate that nanoscale conjugates containing Boswellate and glycurrhetinate moieties in combination with antioxidant peptide moieties can impact the expression of pro-inflammatory cytokines such as IL-6 which is promising.

## 4. Conclusions

In this study, we report the investigation, preparation and characterization of nanoconjugates of known antioxidant peptides YYIVS, GSGGL and MPDAHL with the natural products Boswellate and glycyrrhetinate. Molecular docking studies revealed that the conjugates had a strong binding affinity toward α-Synuclein fibrils which are implicated in PD. Those studies were further validated through molecular dynamics simulations which showed the formation of stable complexes making critical interactions with residues of the pre-NAC and NAC region of α-Syn fibrils. The conjugates were then synthesized and self-assembled into nanovesicles, nanospheres or nanofibers and their binding interactions with α-Syn fibrils were explored through FTIR and CD spectroscopy. Furthermore, the nanoconjugates were found to be generally non-cytotoxic, reduced IL-6 expression in microglial cells and enhanced the uptake of α-Syn in microglial cells. These results open the possibility to develop more fine-tuned nanoscale biomaterials from natural products for diagnostic or therapeutic applications. Our results may be important to set up a further experimental investigation in vivo and potentially lead the path for the design and further functionalization of these nanomaterials for use as possible imaging materials or for potentially targeted therapeutics. Further studies on the detailed mechanism of cellular pathways are ongoing and will be reported separately.

## Figures and Tables

**Table 1:** Binding affinities of α-Syn fibrils toward designed conjugates and peptides.

Conjugate/Peptide	Binding Affinity (kcal/mol)
Boswellate-YYIVS (BA-Y)	−9.7
Boswellic-GSGGL (BA-G)	−10.2
Boswellate-MPDAHL (BA-M)	−9.2
Glycyrrhetinate-YYIVS (GH-Y)	−10.2
Glycyrrhetinate-GSGGL (GH-G)	−10.2
Glycyrrhetinate-MPDAHL (GH-M)	−9.4
YYIVS	−7.9
GSGGL	−6.2
MPDAHL	−7.8

**Table 2:** PLIP analysis showing interacting residues and bond distances of conjugates and peptides with α-Syn fibrils.

Type of Interaction	BA-Y Res: Dist	BA-G Res: Dist	BA-M Res: Dist	GH-Y Res: Dist	GH-G Res: Dist	GH-M Res: Dist	YYIVS Res: Dist	GSGGL Res: Dist	MPDAHL Res: Dist
Hydrophobic	T54: 3.42 A° T54: 3.69 A° T54: 3.78 A° A56: 3.71 A° A56: 3.66 A° T59: 3.96 A° T59: 3.75 A° T75: 3.89 A°	T54: 3.50 A° T54: 3.67 A° T54: 3.86 A° T54: 3.70 A° T54: 3.96 A° A56: 3.68 A° A56: 3.87 A° T59: 3.53 A°	T54: 3.14 A° T54: 3.82 A° A56: 3.82 A° A56: 2.49 A° T59: 3.84 A° T59: 3.64 A° E61: 3.73 A° T75: 3.84 A°	T54: 3.68 A° T54: 3.49 A° A56: 3.59 A° A56: 3.53 A° T59: 3.85 A° T75: 3.62 A°	A56: 3.47 A° A56: 3.34 A° A56: 3.42 A° T59: 3.62 A°	T54: 3.9 A° A56: 3.7 A°	T54: 3.7 A° T54: 3.6 A° A56: 3.5 A° T59: 3.8 A° T59: 3.87 A° T59: 3.70 A°	T54: 3.96 A° A56: 3.9 A° A56: 3.57 A°	T54: 3.46 A°
H-bonds	T59: 2.33 A° T59: 2.49 A° T59: 2.35 A° E61:2.48 A° G73:2.95 A° V74:2.73 A° T75:2.09 A° T75:1.76 A° T75:2.26 A° T75:2.42 A°	T59: 2.10 A° T59: 2.62 A° T59: 2.53 A° T59: 2.22 A° G61: 2.22 A° G73: 3.72 A° V74: 2.84 A° T75: 2.63 A°	T59: 2.28 A° G61: 2.05 A° G61: 3.10 A° G73: 3.62 A° G73: 2.87 A° G73: 3.11 A° V74: 3.74 A° T75: 3.07 A° T75: 2.26 A° T75: 2.04 A°	T59: 3.63 A° E61: 1.99 A° E61: 2.37 A° T72: 2.51 A° T72: 2.66 A° T73: 2.97 A° T73 2.63 A° V74: 3.62 A° V74: 3.46 A° T75: 3.41 A° T75: 3.20 A°	T59: 2.27 A° T59: 2.12 A° T59: 3.37 A° E61: 2.03 A° G73: 2.83 A° V74: 2.63 A° T75: 2.41 A° T75: 2.12 A° T75: 2.39 A° T75: 2.25 A°	T59: 3.15 A° T59: 3.16 A° T59: 2.84 A° T59: 2.61 A° E61: 3.05 A° G73: 2.93 A° G73: 3.00 A° V74: 3.44 A° V74: 3.04 A°	T59: 2.45 A° E61: 2.75 A° E61: 3.21 A° G73: 3.37 A° G73: 3.37 A° G73: 2.11 A° T75: 2.11 A° T75: 3.26 A°	T59: 3.44 A° T59: 2.74 A° E61: 2.62 A° E61: 3.38 A° G73: 2.68 A° G73: 2.43 A° V74: 3.30 A° T75: 2.55 A° T75: 3.31 A° T75: 2.12 A°	T59: 3.33 A° E61: 3.05 A° E61: 2.28 A° G73: 2.27 A° V74: 2.69 A° T75: 2.69 A° T75: 2.46 A°

**Table 3:** Comparison of secondary structural elements obtained from CD spectra.

Nanoconjugate with ASyn	% Helix	% Antiparallel β-Sheets	% Parallel β-Sheets	% Turns	% Others (Disordered)
BA-Y					
1 h	22.2	62.3	0	5.7	9.7
24 h	54.5	30.3	15.2	0	0
48 h	0	17.1	0	19.5	63.4
BA-M					
1 h	5.5	47.5	0	20.7	26.3
24 h	9.3	16.8	17.3	12.4	44.1
48 h	0	41.8	0	14.8	43.3
BA-G					
1 h	0	45.1	0	17.9	36.9
24 h	0	22.4	8	17.9	51.8
48 h	0	42.0	0	15.1	42.9
GH-Y					
1 h	0	46.3	0	15.2	38.5
24 h	2.9	30.1	0	14.3	52.8
48 h	20.8	19.1	25.0	10.7	24.4
GH-M					
1 h	3.7	23.2	11.3	13.8	48.0
24 h	9.1	23.2	0	15.5	52.2
48 h	0	37.6	0	15.0	47.4
GH-G					
1 h	0	41.7	0	15.8	42.5
24 h	3.1	29.3	0	14.5	53.0
48 h	25.6	31.8	17.0	0.6	24.9
Unbound α-Syn					
24 h	0	69.5	0	10.4	20.1

## Data Availability

The original contributions presented in this study are included in the article and in Supplementary Materials.

## Supplementary Materials

The following supporting information can be downloaded at https://www.mdpi.com/article/10.3390/biomimetics10020082/s1, Figure S1: Comparison of (a) RMSD and (b) RMSF plots of the three peptides YYIVS; MPDAHL and GSGGL upon complexation with α-Syn fibrils; Table S1: Comparison of binding affinities of additional four amyloidogenic proteins with the designed conjugates.

## Abbreviations

The following abbreviations are used in this manuscript:

BA	Boswellate
GH	Glycyrrhetinate
BA-Y/GH-Y	Boswellate/glycyrrhetinate conjugate with YYIVS
BA-G/GH-G	Boswellate/glycyrrhetinate conjugate with GSGGL
BA-M/GH-M	Boswellate/glycyrrhetinate conjugate with MPDAHL
