# Supplementary material for: Design and Development of Natural-Product-Derived Nanoassemblies and Their Interactions with Alpha Synuclein
Source: Biomimetics (Basel). 2025 Jan 28;10(2):82. doi: 10.3390/biomimetics10020082 (PMC11852371; doi:10.3390/biomimetics10020082)
Supplement: Supplementary file 1 [file biomimetics-10-00082-s001.zip › biomimetics-3421221-supplementary.pdf]

SUPPLEMENTARY INFORMATION

Design and Development of Natural Product Derived Nanoassemblies and their Interactions with Alpha Synuclein.

Ipsita A. Banerjee [1*], Amrita Das [1†], Mary A. Biggs[1†], Chau Anh N. Phan[1†] Liana R. Cutter[1] and Alexandra R. Ren[1]

Department of Chemistry and Biochemistry, Fordham University, 441 East Fordham Road, Bronx, New York 10458, USA

[†] *Equal contributors.*

Table S1. Comparison of binding affinities of additional four amyloidogenic proteins with the designed conjugates.

Conjugate/ Peptide	Binding Affinity (kcal/mol)	Binding Affinity (kcal/mol)	Binding Affinity (kcal/mol)	Binding Affinity (kcal/mol)
	Tau Paired helical filament (7UPG)	Amyloid Beta Fibril Type B (8EZE)	Amyloid fibril structure of islet amyloid peptide (6Y1A)	Infectious Prion Fibrils (7LNA)
Boswellate-YYIVS (BA-Y)	-8.2	-8.7	-7.3	-8.5
Boswellic-GSGGL (BA-G)	-5.7	-8.2	-6.8	-8.0
Boswellate-MPDAHL (BA-M)	-6.9	-8.2	-7.5	-7.4
Glycyrrhetinate-YYIVS (GH-Y)	-7.4	-9.2	-7.4	-7.3
Glycyrrhetinate-GSGGL (GH-G)	-5.9	-7.7	-7.1	-8.1
Glycyrrhetinate-MPDAHL (GH-M)	-6.6	-7.9	-6.9	-6.7

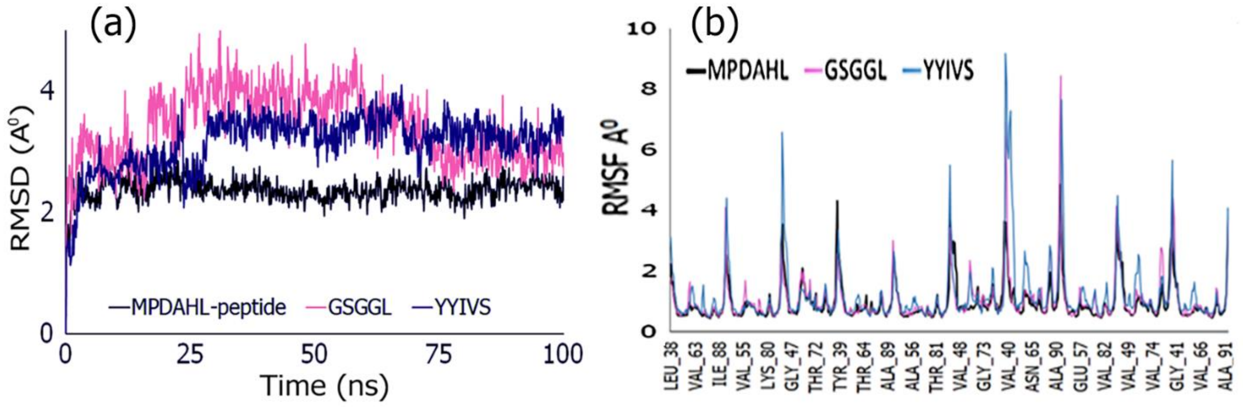

Figure S1. Comparison of (a) RMSD and (b) RMSF plots of the three peptides YYIVS; MPDAHL and GSGGL upon complexation with α-Syn fibrils.
